# Tortuosidade Coronariana como um Novo Fenótipo para Isquemia sem Doença Arterial Coronariana

**DOI:** 10.36660/abc.20220826

**Published:** 2022-11-23

**Affiliations:** 1 Zagazig University – Cardiology Zagazig Egito Zagazig University – Cardiology, Zagazig – Egito

**Keywords:** Doença da Artéria Coronariana/complicações, Isquemia Miocárdica, Aterosclerose, Dislipidemias, Transtornos do Metabolismo do Cálcio/complicações, Fatores de Risco, Diagnóstico por Imagem/métodos

A doença arterial coronariana constitui uma grande carga em muitos países. Em muitos casos, a detecção de isquemia coronariana por imagem não invasiva pode não se correlacionar com a presença de estenose coronariana significativa. Assim, surgiu o termo “Isquemia com artéria coronária não obstrutiva (INOCA)”. Muitas teorias foram propostas para tal fenômeno. A tortuosidade coronariana (TCor) é uma dessas etiologias que se mostrou associada à aterosclerose subclínica e aumento do escore de cálcio coronariano.^1^ Além disso, a TCor está associada aos mesmos fatores de risco de isquemia, como tabagismo, idade avançada, hipertensão arterial e dislipidemia.^2^ TCor foi pensada para ser um fenômeno; entretanto, a associação desse fenômeno com múltiplas doenças cardiovasculares aumenta seu impacto clínico. Li et al.,^3^ verificaram que pacientes hipertensos com TCor apresentam maior incidência de infarto lacunar.^3^ Turgut et al.,^4^ concluíram que a TCor pode indicar relaxamento ventricular esquerdo prejudicado.^4^ Dogdus et al.,^5^ comprovaram que a TCor afeta negativamente a função ventricular esquerda avaliada por parâmetros de deformação 3D com considerável depressão da deformação longitudinal do miocárdio.^5^

Neste estudo de Estrada et al.,^6^ os pesquisadores encontraram uma associação altamente significativa entre TCor e isquemia. A presença de isquemia nos territórios com TCor foi mais frequente do que naqueles sem TCor (67% versus 28% (p<0,0001)) detectada pela cintilografia de perfusão miocárdica (CPM). Este estudo analisou as características específicas da tortuosidade quanto à presença de isquemia miocárdica. Eles descobriram que o número de ângulos de curvatura detectados na sístole durante a angiografia coronária está associado a um alto risco de isquemia miocárdica (p = 0,021). Alguns estudos anteriores consideraram a geometria da TCor e sugeriram índices para graduar tal tortuosidade dependendo principalmente do grau de angulação ou flexão.^7-9^

Várias teorias foram propostas para explicar o mecanismo pelo qual a TCor pode precipitar isquemia. A TCor pode causar disfunção microcirculatória ao reduzir as pressões de enchimento distais e o fluxo sanguíneo. Isso pode ser devido a forças de cisalhamento em artérias tortuosas que podem perturbar a dinâmica do fluxo.^10^ Outros sugeriram que a simples degeneração da camada de elastina do vaso aterosclerótico pode levar à TCor.^9^ Além disso, a TCor é considerada por alguns pesquisadores como um achado comum em pacientes idosos e hipertensos com hipertrofia ventricular esquerda devido ao alongamento e dilatação das coronárias em um espaço limitado dos sulcos coronários levando à flexão ou dobramento das artérias.^11^

Além das teorias mecânicas e hemodinâmicas acima mencionadas, alguns pesquisadores estabeleceram uma teoria inflamatória como mecanismo de aterosclerose no caso de TCor. Li et al.,^3^ propuseram um papel para a reação inflamatória evidenciada por altos níveis de PCR que foram encontrados associados à TCor. No mesmo contexto, Naguib et al.,^12^ estudaram a associação entre TCor em pacientes sem lesões coronarianas e alta contagem de monócitos a baixa relação HDL-C (MHR) como marcador de inflamação e estresse oxidativo. Eles descobriram que o TCor tem uma relação significativa com a MHR, que agora é considerada um marcador prognóstico para muitas doenças cardiovasculares. Além disso, Cerit et al.,^13^ verificaram que o plaquetócrito, importante para inflamação e trombose, estava independentemente associado à TCor.

Este estudo de Estrada et al.,^6^ estudou a relação entre a isquemia induzida por TCor e o ramo coronariano acometido. Eles encontraram uma associação significativa entre TCor e isquemia na ACX e ACD, mas essa associação não foi significativa na ADAE. Além disso, o grau de TCor (evidenciado por ângulos de curvatura consecutivos e o número de ângulos de curvatura) teve uma associação significativa com isquemia apenas em ACX. Acho que este é o primeiro estudo a analisar a relação entre TCor e isquemia em territórios coronarianos individuais.

No entanto, ainda há controvérsia sobre a real relação entre TCor e isquemia coronariana. Apesar dos achados anteriores, há alguns votos contra o papel do TCor na aterosclerose. Li et al.,^2^ não encontraram uma correlação significativa entre TCor e escore de cálcio ou estenose de diâmetro na análise multivariada. No entanto, essa associação existia entre TCor e escore de cálcio moderado entre as mulheres.^2^ Na mesma direção, Khosravani-Rudpishi et al.,^14^ encontraram que vasos tortuosos tinham menor probabilidade de estenose significativa da artéria coronária e menor escore de Gensini.^14^ Além disso, foram encontrados dados controversos sobre a gravidade do TCor e sua associação com doença arterial coronariana significativa. Enquanto Hassan et al.,^8^ verificaram que a tortuosidade grave está associada a um risco aumentado de isquemia, ao contrário, Groves et al.,^7^ descobriram que pacientes com tortuosidade coronariana grave tiveram uma incidência significativamente menor de estenose significativa da artéria coronária na angiografia coronária.^7^

Esses dados aparentemente controversos poderiam ser justificados pela hipótese de que os vasos tortuosos podem desenvolver alterações ateroscleróticas e calcificadas; no entanto, essas alterações estão longe das próprias curvas.

Acho que precisamos de mais estudos para desenvolver um índice de tortuosidade mais válido e correlacionar diferentes graus de TCor com modalidades funcionais de avaliação de isquemia como FFR e iFR.

## References

[1] El Tahlawi M, Sakrana A, Elmurr A, Gouda M, Tharwat M (2016). The relation between coronary tortuosity and calcium score in patients with chronic stable angina and normal coronaries by CT angiography. Atherosclerosis.

[2] Li M, Wang ZW, Fang LJ, Cheng SQ, Wang X, Liu NF (2022). Correlation analysis of coronary artery tortuosity and calcification score. BMC Surg.

[3] Li Y, Nawabi AQ, Feng Y, Ma G, Tong J, Shen C (2018). Coronary tortuosity is associated with an elevated high-sensitivity C-reactive protein concentration and increased risk of ischemic stroke in hypertensive patients. J Int Med Res.

[4] Turgut O, Yilmaz A, Yalta K, Yilmaz BM, Ozyol A, Kendirlioglu O (2007). Tortuosity of coronary arteries: An indicator for impaired left ventricular relaxation?. Int J Cardiovasc Imaging.

[5] Dogdus M, Demir E, Cinar CS, Gurgun C (2020). Coronary tortuosity affects left ventricular myocardial functions: a 3D-speckle tracking echocardiography study. Int J Cardiovasc Imaging.

[6] Estrada A, Sousa AS, Mesquita CT, Villacorta H (2022). Coronary Tortuosity as a New Phenotype for Ischemia without Coronary Artery Disease. Arq Bras Cardiol.

[7] Groves SS, Jain AC, Warden BE, Gharib W, Beto RJ (2009). Severe coronary tortuosity and the relationship to significant coronary artery disease. W V Med J.

[8] Hassan AKM, Abd-El Rahman H, Hassan SG, Ahmed TAN, Youssef AAA (2018). Validity of tortuosity severity index in chest pain patients with abnormal exercise test and normal coronary angiography. Egypt Heart J.

[9] Jakob M, Spasojevic D, Krogmann ON, Wiher H, Hug R, Hess OM (1996). Tortuosity of coronary arteries in chronic pressure and volume overload. Cathet Cardiovasc Diagn.

[10] Zegers ES, Meursing BTJ, Zegers EB, Ophuis AJMO (2007). Coronary tortuosity: a long and winding road. Neth Heart J.

[11] Hutchins GM, Bulkley BH, Miner MM, Boitnott JK (1977). Correlation of age and heart weight with tortuosity and caliber of normal human coronary arteries. Am Heart J.

[12] Naguib TA, Farag ESM, El Tahlawi MAA, Shawky AF (2022). Relationship between Monocyte to High Density Lipoprotein Cholesterol Ratio and Coronary Artery Tortuosity. Egypt J Hosp Med.

[13] Cerit L, Cerit Z (2017). Relationship between coronary tortuosity and plateletcrit coronary tortuosity and plateletcrit. Cardiovasc J Afr.

[14] Khosravani-Rudpishi M, Joharimoghadam A, Rayzan E (2018). The significant coronary tortuosity and atherosclerotic coronary artery disease; What is the relation?. J Cardiovasc Thorac Res.

