# Supplementary material for: Tortuosidade Coronariana como um Novo Fenótipo para Isquemia sem Doença Arterial Coronariana
Source: Arq Bras Cardiol. 2022 Nov 23;119(6):891–2. [Article in Portuguese] doi: 10.36660/abc.20220826 (PMC9814803; doi:10.36660/abc.20220826)
Supplement: Translation

# Translation: Coronary Tortuosity as a New Phenotype for Ischemia without Coronary Artery Disease

**Affiliations:** 1 Zagazig Egypt Zagazig University – Cardiology, Zagazig – Egypt

**Keywords:** Coronary Artery Disease/complications, Myocardial Ischemia/complications, Atherosclerosis, Dyslipidemias, Calcium Metabolism Disorders/complications, Risk Factors, Diagnostic Imaging/methods

Coronary artery disease constitutes a great burden in many countries. In many cases, the detection of coronary ischemia by non-invasive imaging may not correlate with the presence of significant coronary stenosis. Therefore, the term “Ischemia with non-obstructive coronary artery (INOCA)” has emerged. Many theories have been proposed for such a phenomenon. Coronary tortuosity (CorT) is one of these etiologies that was found to be associated with subclinical atherosclerosis and increased coronary artery calcium score.^1^ Besides, CorT is associated with the same risk factors of ischemia, such as smoking, old age, arterial hypertension and dyslipidemia.^2^ CorT was thought to be a phenomenon; however, the association of this phenomenon with multiple cardiovascular disorders augments its clinical impact. Li et al.^3^ found that Hypertensive patients with CorT have more incidence of lacunar infarct.^3^ Turgut et al.^4^ concluded that CorT might indicate impaired left ventricular relaxation.^4^ Dogdus et al.^5^ proved that CorT negatively affects left ventricular function evaluated by 3D strain parameters with considerable depression of longitudinal deformation of the myocardium.^5^

In this study by Estrada et al.,^6^ the investigators found a highly significant association between CorT and ischemia. The presence of ischemia in territories with CorT was more frequent than those without CorT (67% versus 28% (p<0.0001)) detected by myocardial perfusion imaging (MPI). This study analyzed the specific features of tortuosity concerning the presence of myocardial ischemia. They found that the number of bend angles detected in systole during coronary angiography is associated with a high risk of myocardial ischemia (p = 0.021). Some previous studies considered the geometry of CorT and suggested indices for grading such tortuosity depending mainly on the degree of angulation or bending.^7-9^

Multiple theories were proposed to explain the mechanism that CorT might precipitate ischemia. CorT may cause microcirculatory dysfunction by reducing distal filling pressures and blood flow. This may be due to shearing forces in tortuous arteries that could disturb flow dynamics.^10^ Others suggested that simple degeneration of the elastin layer of the atherosclerotic vessel may lead to CorT.^9^ Furthermore, CorT is considered by some investigators as a common finding in elderly and hypertensive patients with left ventricular hypertrophy due to elongation and dilatation of coronaries in a limited space of coronary sulci leading to bending or folding of the arteries.^11^

In addition to the abovementioned mechanical and hemodynamic theories, some investigators settled an inflammatory theory as a mechanism of atherosclerosis in the case of CorT. Li et al.^3^ proposed a role for inflammatory reaction evidenced by high CRP levels that were found to be associated with CorT. In the same context, Naguib et al.^12^ studied the association between CorT in patients without coronary lesions and high monocyte count to low HDL-C ratio (MHR) as a marker of inflammation and oxidative stress. They found that CorT has a significant relationship with MHR, which is now considered a prognostic marker for many cardiovascular diseases. Furthermore, Cerit et al.^13^ found that plateletcrit, which is important for inflammation and thrombosis, was independently associated with CorT.

This study by Estrada et al.^6^ studied the relationship between CorT-induced ischemia and the affected coronary branch. They found a significant association between CorT and ischemia in the LCX and RCA, but this association was not significant in LAD. Furthermore, the degree of CorT (evidenced by consecutive bend angles and the number of bend angles) had a significant association with ischemia only in LCX. I think this is the first study to analyze the relationship between CorT and ischemia in individual coronary territories.

However, there is still controversy regarding the actual relationship between CorT and coronary ischemia. Despite the previous findings, there are some votes against the role of CorT in atherosclerosis. Li et al.^2^ failed to find a significant correlation between CorT and calcium score or diameter stenosis on multivariable analysis. However, this association existed between CorT and moderate calcium score among women.^2^ In the same direction, Khosravani-Rudpishi et al.^14^ found that tortuous vessels had a lower probability of coronary artery significant stenosis and a lower Gensini score.^14^ Furthermore, controversial data were found regarding the severity of CorT and its association with significant coronary artery disease. While Hassan et al.^8^ found that severe tortuosity is associated with an increased risk of ischemia, on the contrary, Groves et al.^7^ found that patients with severe coronary tortuosity had a significantly lower incidence of significant coronary artery stenosis in coronary angiography.^7^

These apparently controversial data could be justified by hypothesizing that the tortuous vessels may develop atherosclerotic and calcific changes; however, these changes are away from the bends themselves.

I think we need further studies to develop a more valid tortuosity index and to correlate different grades of CorT with functional modalities of assessment of ischemia such as FFR and iFR.
